# Case of Impending Diabetic Coma

**Published:** 1893-06

**Authors:** H. McHatton

**Affiliations:** Macon, Ga.


### CASE OF IMPENDING DIABETIC COMA.

H. McHATTON, M. D., MACON, GA.

Mr. C., 64 years of age, diabetic of 12 years standing. Has been under treatment all the time, having spent years in Europe, and several seasons at Carlsbad. Has just returned from a trip to Cuba and Florida, and has been unusually free with his diet. I saw him first on the morning of March 15th. His general nutrition was good. Found him suffering with a severe gastro-intestinal irritation, vomiting continually and purging. Pulse 100, respiration 30, and labored, tongue dry. Decided pain in the left hypocondrium, drowsy and weak, anorexia complete, slight jaundice showing on the conjunctivæ. Gave him a mixture of aromatic spirits of ammonia and subnitrate of bismuth to quiet stomach as soon as possible. P.M.—Slight amelioration of condition of stomach, other symptoms worse.

16th, P. M.—Passed 16 oz. urine in the past 24 hours, sugar abundant, albumen by Heller's test, specific gravity, 1,015 ; a few hyaline casts, one or two to the slide; no more vomiting, pulse 120, respiration 40, hebetude and præcordial pain increased, jaundice complete ; inf. digitalis ℨi. potass. acet gr. 15, every 2 hours with directions to drink as much lithia water and milk as he could contain. P. M.—General symptoms worse, hebetude increased, pulse 140, respiration 50 ; condition alarming. Inf. digitalis and potass. acet every 4 h., nitro-glycerine, gr. 1-100 every 4 h. alternating, continue ingestion of large quantity of liquid.

17th, A. M.—Pulse 120, respiration 35, jaundice increasing, but every other symptom better; passed 4 pints urine, same in every respect as that of 16th.

18th, A. M.—Pulse 100, respiration 30 ; about 5 pints urine ; less sugar, less albumen, specific gravity, 1,015.

19th, A. M.—Pulse 80, respiration 20 ; much better in every respect excepting jaundice ; about 6 pints urine, specific gravity, 1,014 ; no casts, no sugar, no albumen, neither of which were found subsequently. P. M.—Pulse 80, respiration 22; increased interval of medicine to four and one-half hours.

20th, A. M.—Pulse 80, respiration 20 ; up and dressed. P. M.—Pulse 80, respiration 18; increased intervals of medicine to five hours ; jaundice diminishing.

21st, A. M.—Pulse 78, respiration 18 ; between 6 and 7 pints of urine; increased intervals of medicine to six hours.

22d. Feels as well as usual, and insists on starting on a two day's railroad journey, which I understand was made with comparative comfort.

That, gentlemen, covers the history treatment and result in this case. In looking it over closely you will see that there were several unusual problems to be solved.

First. There was the gastro-intestinal irritation and jaundice, which in this climate is usually attributed to inactivity of the liver.

Second. Small amount of urine, low gravity and a few casts, almost pathognomonic of chronic Brights, if taken alone.

Third. The sugar, previous history, rapid pulse and respiration, expiration being prolonged, anorexia, hebetude, pain in left hypocondrium, dry tongue, thirst and odor, indicating impending diabetic coma, which was my diagnosis. I disregard the jaundice entirely as being probably catarrhal. The casts being so few in number as of possibly no import and regarded the albumen as a distinct symptom of impending coma in this type of cases. As it is asserted by good authority that no case of recovery from diabetic coma is known, our only chance is to prevent that condition.

Late observations tend to show that flooding the patient through the stomach with liquids is our sheet anchor. The amount of liquid taken in this case being enormous the idea of using diuretics that are at the same time cardiac stimulants, I have never seen mentioned, but it seems to have had a most beneficial effect in this special case.

The suggestion of large amounts of liquid used in this manner is due I think to Dr. Reynolds, who reports two cases of recovery similar to this one. The question has been raised as to whether the beneficial results are due to the dilution or increased elimination of the poison. In the present case the latter seems to have been an important factor, as the amount of urine increased from 16 to 100 ounces.

I regret that other urgent cases rendered it impossible for me to make a complete quantitative analysis of sugar and albumen and note other conditions of the urine such as acetone reaction in this case.

---

The preparations of "PEPSIN," made by Robinson-Pettet Co., are endorsed by many prominent physicians. We recommend a careful perusal of the advertisement of this well-known manufacturing house. (See advertisement.)

